# Lead Dependent Tricuspid Valve Dysfunction-Risk Factors, Improvement after Transvenous Lead Extraction and Long-Term Prognosis

**DOI:** 10.3390/jcm11010089

**Published:** 2021-12-24

**Authors:** Anna Polewczyk, Wojciech Jacheć, Dorota Nowosielecka, Andrzej Tomaszewski, Wojciech Brzozowski, Dorota Szczęśniak-Stańczyk, Krzysztof Duda, Andrzej Kutarski

**Affiliations:** 1 Department of Physiology, Patophysiology and Clinical Immunology, Collegium Medicum of Jan Kochanowski University, 25-369 Kielce, Poland; 2 Department of Cardiac Surgery, Świętokrzyskie Centrum of Cardiology, 25-736 Kielce, Poland; 3 2nd Department of Cardiology, Zabrze, Faculty of Medical Science in Zabrze, Medical University of Silesia in Katowice, 41-800 Zabrze, Poland; wjachec@interia.pl; 4 Department of Cardiology, The Pope John Paul II Province Hospital of Zamość, 22-400 Zamosc, Poland; dornowos@wp.pl; 5 Department of Cardiology, Medical University of Lublin, 20-059 Lublin, Poland; benecho2008@gmail.com (A.T.); brzozo@wp.pl (W.B.); dorotasstanczyk@gmail.com (D.S.-S.); 6 Department of Cardiac Surgery, Masovian Specialistic Hospital of Radom, 26-617 Radom, Poland; kadeder@gmail.com (K.D.); a_kutarski@yahoo.com (A.K.)

**Keywords:** lead dependent tricuspid valve dysfunction, transvenous lead extraction, improvement of tricuspid valve function, prognosis

## Abstract

Background: Lead-related tricuspid valve dysfunction (LDTVD) has not been studied in a large population and its management remains controversial. Methods: An analysis of the clinical data of 2678 patients undergoing transvenous lead extraction (TLE) in years 2008–2021 was conducted, with a separate group of 119 patients with LDTVD. Potential risk factors for LDTVD, improvement in valve function, and long-term prognosis after TLE were assessed. Results: LDTVD was diagnosed in 4.44% of patients referred for lead extraction due to different reasons. The most common mechanism of LDTVD was propping upward or clamping down the leaflet by the lead (85.71%). The probability of LDTVD was higher in female sex, patients with valvular heart disease, atrial fibrillation, heart failure, large right ventricle and high pulmonary artery systolic pressure, the presence of only pacing lead, and in case of collision of the lead with tricuspid valve and adhesion of the lead to the heart structures. The prognosis of patients with LDTVD was worse, however, patients with improved valve function after TLE showed a significantly better long-term survival. Conclusions: Lead dependent tricuspid valve dysfunction is a potentially serious condition that requires thorough diagnostics and thoughtful management. The risk factors for LDTVD are primarily related to the course of the lead and its adhesion to the heart structures. Improvement of tricuspid valve function after TLE is observed in 35.29% of patients Patients with LDTVD have a worse long-term survival, but the improvement in valve function following TLE contributes to a significant reduction in mortality.

## 1. Introduction

The problem of tricuspid valve dysfunction after implantation of an endocardial lead was considered from the beginning of pacing era [1,2]. In the following years, there were divergent reports on lead dependent tricuspid valve dysfunction (LDTVD). Most studies confirm the deterioration of tricuspid valve (TV) function in patients with cardiac implantable electronic devices (CIED) [2,3,4,5,6,7,8,9,10,11], but some reports contradict the presence of this phenomenon [12,13,14]. Previous researches were often based on small populations with a short follow-up period and sometimes did not include pre-implantation echocardiography. Currently, there have been several reports assessing the occurrence of LDTVD in a large group of patients and a longer follow-up period after CIED implantation [15,16,17]. All these studies confirm the incidence of LDTVD increasing over time and look for potential risk factors for worsening of tricuspid valve function. The present study is, to our knowledge, based on the largest population to date and includes an analysis of many factors that may influence the development of LDTVD and an assessment of the possibility of improving tricuspid valve function and long term survival after transvenous lead extraction.

## 2. Materials and Methods

### 2.1. Patient Population

A total of 3500 patients who underwent transvenous lead extraction (TLE) procedures by one key operator at three high volume centers between June 2008 and September 2021 were included into this study. All information regarding the patient and the procedure was entered into the computer database on a current basis. Patients without complete echocardiographic findings before and after TLE were excluded. Finally, a total of 2678 patients were enrolled in this study. Patients were divided into two main groups: Group 1 consisted of 119 (4.44%) of patients with lead dependent tricuspid valve dysfunction and Group 2 consisted of 2559 patients without LDTVD. Additionally, an analysis of two LDTVD subgroups was performed—with and without improvement in tricuspid valve function after TLE (Supplementary File).

### 2.2. Baseline Parameters

Demographic data, comorbidities and history of pacing were analyzed. The incidence of coronary heart disease, cardiomyopathy, valvular heart diseases and other comorbidities: hypertension, atrial fibrillation, congestive heart failure, diabetes, renal failure, long-term anticoagulation, and Charlson’s comorbidity index, was compared in the studied groups. The comparison of indication-related, system-related and history of pacing related factors in patients with and without LDTVD was also conducted including frequency of occurrence: lead -related infective endocarditis (LRIE), local, pocket infection and non-infective indications for TLE, type of implanted device, number of implanted leads, location of the tip of the lead, abnormal elongated loop of the lead and dwell time of the leads. The complexity of procedures, effectiveness of TLE, presence of complications and short-, medium- and long-term mortality after TLE were also compared in the study groups.

### 2.3. Echocardiography

Transthoracic echocardiography (TTE) was performed in all patients before and after transvenous lead extraction. Transesophageal echocardiography (TEE) was performed before and after TLE in all of patients undergoing TLE in years 2008–2015. A total of 90% of TLE procedures have been continuously monitored by TEE since 2016 with the precise assessment of TV function before, during and after procedure.

TTE and TEE in our series was performed using Philips iE33 (Phillips Healthcare, Andover, MA, USA) and GE Vivid S 70 GE Healthcare machines (General Electric Company, Boston, MA, USA) equipped with X7-2t Live 3D (Phillips Healthcare, Andover, MA, USA) or 6VT-D probes (General Electric Company, Boston, MA, USA). All recordings were archived and carefully assessed by two experienced cardiologists who were blinded to the clinical data.

Tricuspid regurgitation (TR) severity was graded semi-quantitatively using colored and continuous wave Doppler data using a multi-parametric approach [18,19] including valve morphology, colour flow jet, continuous wave signal of the jet, vena contracta width, and were categorized in three groups: no, trace or mild (0, 1st, 2nd Grades), moderate (3rd Grade), and severe (4th Grade). Lead removal associated improvement of TR was defined by reduction of TR from severe to moderate/mild or from moderate to mild comparing echocardiography studies before and after TLE. Pulmonary artery systolic pressure (PASP) was calculated as the sum of the tricuspid jet gradient (assessed by Doppler) and right atrial pressure.

### 2.4. Definitions

Lead dependent tricuspid valve dysfunction was defined as significant or severe tricuspid regurgitation (or stenosis) resulting from the documented influence of the lead on the valve leaflets or chordae tendinae. LDTVD was recognized based on the visualization of one of the triggering mechanisms of TR: propping the leaflet by the lead or impingement of the leaflet by the lead or presence of the loop of lead irritating the TV or perforation of the leaflet with the lead.

Lead extraction procedure was defined according to the most recent guidelines on the management of lead-related complications (HRS 2017 and EHRA 2018) [20,21]. Indications for TLE and type of periprocedural complications were defined according to the 2017 HRS Expert Consensus Statement on Cardiovascular Implantable Electronic Device Lead Management and Extraction [20].

### 2.5. Transvenous Lead Extraction Procedure

Most TLE procedures were performed using nonpowered mechanical systems such as Byrd polypropylene dilator sheaths (Cook Medical, Leechburg, PA, USA) if only possible via the implant vein. If technical difficulties arose, alternative venous approaches or additional tools such as Evolution (Cook Medical, Leechburg, PA, USA), TightRail (Spectranetix, Sunnyvale, CA, USA), lassos (Multi-Snare^®^Device PFM Medical, Inc. Carlsbad, CA, USA), basket catheters (Cook Medical Inc., Bloomington, IN, USA) were utilized. The excimer laser was not applied.

### 2.6. Indications for Transvenous Lead Extraction in Whole Examined Population of Patients

Main indications for TLE were: 1. infectious complications: local pocket infection, bacteraemia with or without endocarditis, or any combination of these presentations together 2 non-infectious indications including: mechanical lead damage (electric failure), lead dysfunction (exit/entry block, dislodgement, extracardiac pacing, perforation), upgrading, downgrading and another reasons of prevention of lead abandonment-prophylactic indications e.g. atrial fibrillation, overmuch of leads, threatener/potentially threatener lead (free ending, left heart, LDTVD and other (MRI indication, cancer, pain of pocket, loss of indication for pacing/ICD) and recapture venous access (symptomatic occlusion, superior vena cava syndrome, lead replacement/upgrading).

### 2.7. Statistical Analysis

The Shapiro–Wilk test showed that most continuous variables were normally distributed. For uniformity, all continuous variables are presented as the mean ± standard deviation and were compared using “U” Mann–Whitney test. Categorical data are presented as absolute numbers and percentages and were compared using Chi-square test with Yates correction.

Logistic linear regression was applied to identify the variables associated with LDTVD. To the univariable regression analysis were included demographic, clinical, echocardiographic, and CIED related (outside derivate) data which reached the *p* value < 0.1 in the “U” Mann–Whitney or Chi^2^ tests. To the multivariable regression analysis data reached *p* < 0.1 under univariable analysis were included.

The results of the regression analysis were reported as odds ratio with corresponding 95% confidence intervals (CIs).

Survival analysis based on Kaplan–Meier curves and log-rank tests were used to assess the event-free survival between groups of patients separated on the basis of LDTVD presence (divided in to two groups regarding on the TLE impact on the LDTVD).

The results were considered statistically significant if *p* < 0.05. Statistical analysis was performed with Statistica version 13.3 (TIBCO Software Inc., Palo Alto, CA, USA).

### 2.8. Approval of the Bioethics Committee

All patients gave their informed written consent to undergo TLE and use anonymous data from their medical records, approved by the Bioethics Committee at the Regional Chamber of Physicians in Lublin No. 288/2018/KB/VII. The study was carried out in accordance with the ethical standards of the 1964 Declaration of Helsinki.

## 3. Results

### 3.1. Baseline Characteristics

Among 2678 patients referred to TLE, 119 (4.44%) were diagnosed with LDTVD. Patients with LDTVD were referred for lead extraction for various reasons and only 37.82% of LDTVD was the primary indication of TLE. In as far as 62.18% LDTVD is diagnosed occasionally as important but co-existing indication. A common mechanism of LDTVD was propping upward or clamping (drawing) down the leaflet by the lead (85.71%); another mechanism as impingement of the leaflet by the lead presence (irritation and degeneration), perforation of the leaflet with the lead or connection of leaf with the lead with scar were rare (11.76%). In direct echocardiographic evaluation (TEE after procedure), improvement in TV function, defined as perceptible, was found in 27.73% of patients, in 10.92% it showed a significant improvement, while in 61.34% of patients it was not significant. The reduction of TR after TLE was observed in 35.29% of patients: a one-degree reduction in TR was diagnosed in 31.93% of patients, and a 2-degree reduction in 3.36% of patients.

A total of 40 patients out of 119 (33.60%) achieved the criterion of TV plastic, but only 18 (15.13%) were referred for surgery, the rest are under observation (Table 1).

Patients with LDTVD tended to be older at the time of TLE (*p* = 0.087) and more often of the female sex. LDTVD group was characterized by significantly higher incidence of valvular heart disease, presence of valvular implant and severe heart failure (NYHA Class III and IV). Patients with LDTVD were more likely to have permanent atrial fibrillation (AF), renal failure and used long-term anticoagulation (Table 2).

Improvement in tricuspid valve function after TLE was observed in 42 patients with LDTVD, the remaining 77 did not show changes. The reduction in TR after TLE was found more frequently in older patient (in advanced age during implantation and during TLE) and in patients with ischemic heart disease (Table S1—Supplementary File).

### 3.2. Pacing System and TLE-Related Factors

Indications for TLE in the entire study group included infectious complications in 31.52% of patients, non-infectious therapeutic indications in 65.10% and prophylactic indications in 3.40% of patients.

Patients with LDTVD were referred to TLE more frequently due to non-infectious therapeutic indications, less often due to infectious complications.

In terms of kind of implanted system, patients with LDTVD more often had pacemakers (AAI, VVI, DDD, CRT-P mode), less often ICD. Excessive loops of the leads passing through the TV were found more frequently in LDTVD group (20.17% vs. 4.53%; *p* = 0.001), similarly other forms of collision of the lead with TV visible in fluoroscopy (21.01% vs. 0.59%; *p* = 0.001).

The number of leads and number of abandoned leads was comparable in the studied groups.

Mean lead dwell time in the patient before TLE in the whole study group was 95.36 ± 67.10 months and was longer in patients with LDTVD (107.73 ± 68.90 vs. 95.83 ± 66.98 months; *p* = 0.058).

In patients with LDTVD, there was a tendency to more frequent non-apical location of the lead tip (*p* = 0.092) (Table 3).

Improvement of TV function after TLE was visible only in the group of non-infectious prophylactic indications. There was no association between implanted system-related factors and appearance of improvement in TV function after TLE (Table S2—Supplementary File).

Complex (second line) tools such as Evolution, TightRail or lasso catheter were more often required when performing TLE procedures in patients with LDTVD—this was due to more frequent technical problems in this group of patients (10.08% vs. 4.42%; *p* = 0.07). In the LDTVD group, tip or lead fragment retention was more frequent—partial radiological success was observed in 8.40% of patients compared to 3.63% in patients without LDTVD (*p* = 0.014). The effectiveness of TLE and the percentage of major and minor complications did not differ significantly in the studied groups. Long-term mortality after TLE was comparable in the LDTVD group and without lead-dependent tricuspid valve dysfunction (Table 4).

There was no direct relationship between the efficacy of TLE and the improvement in tricuspid valve function after procedure. Patients with improved tricuspid valve function after TLE showed a better long-term survival (Table S3—Supplementary File).

### 3.3. Echocardiographic Findings

Patients with LDTVD more often demonstrated mitral valve insufficiency, greater dimension of the right ventricle (RV) and higher pulmonary systolic pressure (PASP). Left ventricular ejection fraction (LVEF) no differ significantly between compared groups. Significant and severe (Grade 3 and 4) tricuspid regurgitation was more frequently observed in patients with LDTVD (40.34%vs 14.10%; *p* = 0.001 and 47.90% vs. 2.74; *p* = 0.001).

**Figure 1:**
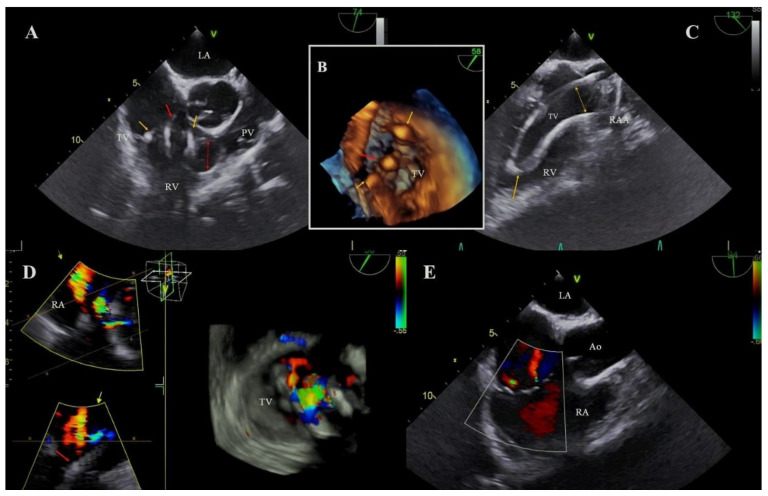
The excessive loop of the lead as a reason of LDTVD. (**A**): The loop of the atrial lead in the tricuspid valve (TV), entering the right ventricular (RV) lumen (yellow arrows). A ventricular lead through the TV forms a loop in the right ventricular outflow tract RVOT (red arrows). TEE 2D. (**B**): Three leads visible in the tricuspid valve, atrial loop of the lead distributed in the commissures (yellow arrows), centrally located ventricular lead (red arrow). TEE 3D. (**C**): After removal of the ventricular lead (TLE), the atrial lead loop was clearly visible. TEE 2D. (**D**): Two jets of a significant tricuspid regurgitation (TR) to the right atrium resulting from the collision of the three leads with the valve leaflets. TEE 3D color doppler. (**E**): The effect after TLE procedure. Two jets of non-significant tricuspid regurgitation remained. TEE 2D.

In the LDTVD group more frequent occurred adhesion of the lead to the walls of the heart, especially to the tricuspid apparatus (14.29% vs. 4.85%; *p* = 0.001) and more often were observed excessively elongated loops of the leads (similarly to fluoroscopy results) (36.13% vs. 17.60%; *p* = 0.001) (Table 5, Figure 1).

There was a trend towards improvement of TV function after TLE in patients with a smaller right ventricular size (*p* = 0.081). The remaining echocardiographic findings did not correlate with a reduction in TV regurgitation after TLE (Table S4—Supplementary File).

### 3.4. Univariate and Multivariate Logistic Regression of Risk Factors for LDTVD

Univariate analysis showed, that factors predisposing to LDTVD were: female gender, valvular heart disease, presence of valvular implant, higher NYHA class, permanent AF, long-term anticoagulation, larger diameter of RV, higher PASP, and strong connective tissue scar connection of the lead with heart structures (TV, RA or RV). There was also a tendency to influence the mean lead dwell time (*p* = 0.062).The presence of only the pacing lead (no HV lead) also increased the probability of LDTVD.

The multivariate analysis confirmed the increased probability of LDTVD in female sex, patients with valvular heart disease, atrial fibrillation, heart failure with high NYHA class, larger RV and higher PASP and in case of collision of the lead with TV and strong connective tissue scar connection of the lead with heart structures especially with RA wall. The presence of only the pacing lead (no HV lead) also increased the probability of LDTVD (Table 6).

**Figure 2:**
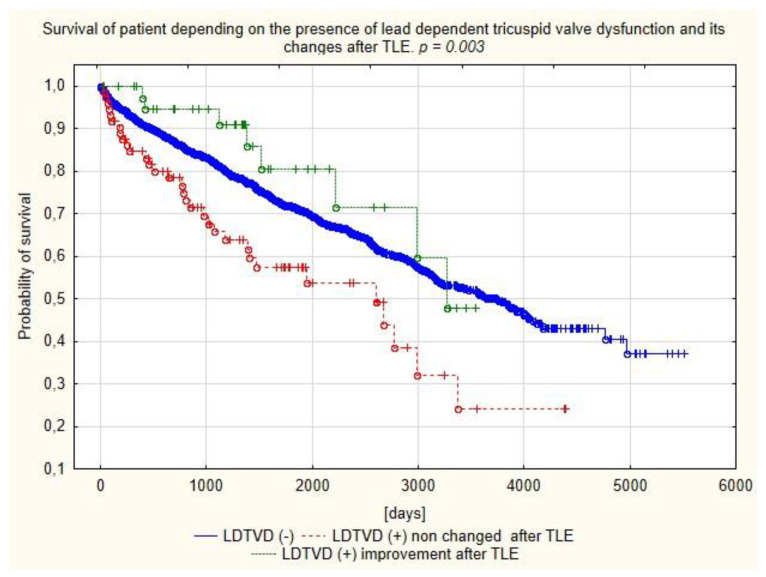
Kaplan–Meier curves of survival depending on the prevalence of LDTVD and changes after TLE.

Analysis of the survival showed better prognosis of patients with LDTVD with improvement of tricuspid valve function after TLE compared to the other LDTVD patients in short, mid and long-term follow up (Figure 2).

## 4. Discussion

The prevalence of lead-dependent tricuspid valve dysfunction is assessed in wide ranges depending on the studied population and the follow-up period after CIED implantation. According to previous studies comparing valve function before and after implantation, deterioration of tricuspid regurgitation is found in 7–45% of patients in Period 1 month to 6.5 years [2,3,4,5,6,7,8,9,10,11,15]. In the present study, the incidence of LDTVD was relatively low—4.44%, however, in the current analysis TV function before and after CIED implantation was not compared, only influence of the lead on TV at various times after implantation (average time was about 8 years) as well as possible reduction of TR and survival after lead extraction procedure.

**Figure 3:**
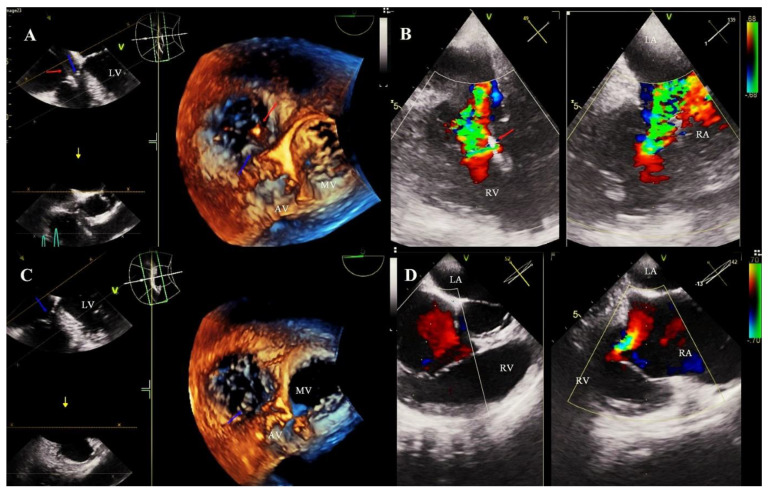
Tricuspid valve dysfunction with significant regurgitation as a result of an unfavorable interaction of the lead with the valve leaflet. Monitoring of the TLE procedure by TEE examination. (**A**): Septal leaflet is clamping down (blue arrow) by a ventricular lead (red arrow) with impaired coaptation of the tricuspid valve TEE 3D. (**B**): Severe tricuspid regurgitation as a consequence of pulling the leaflet. TEE 2D color Doppler valve. (**C**): Imaging after TLE. Improving the mobility of the septal leaflet and the coaptation zone (blue arrow). TEE 3D. (**D**): Significant improvement in the degree of valve regurgitation after extraction of the lead. TEE 2D.

The diagnosis of LDTVD is difficult and requires careful echocardiographic evaluation. It has been shown to be the most common mechanism in the present study were propping upward or clamping (drawing) down the leaflet by the lead (85.7%) (Figure 3). A similar mechanism is presented as leading in the current reports [15,17].

The most frequently reported risk factors for LDTVD are: female sex, the presence of atrial fibrillation, history of open-heart surgery, pre-existing TR, right ventricular dilatation, mitral regurgitation, enlargement of the left atrium, the presence of a high voltage (HV) lead, a greater number of leads passing through the tricuspid orifice, the specific position of the lead in relation to the valve annulus, leaflets, chordae tendinae, the place of pacing in the right ventricle, echocardiographic evidence of leaflet interference and elevated preimplant tricuspid regurgitation pressure gradient [6,7,15,16,17,22,23]. Analysis of the risk factors of LDTVD in the present study also showed a link between the female gender and some clinical factors: valvular heart disease permanent AF and heart failure. These factors do not initially cause LDTVD but are likely to increase the severity of LDTVD and worsen the NYHA class. The present study also showed a significant role for echocardiographic findings. In addition to factors considered in previous reports, such as dilation of the right ventricle and elevated PASP, a higher probability of LDTVD has also been shown in the case of collision of the lead with TV (especially excessive loop) and connective tissue adhesions of the leads with the cardiac structures: TV, RA or RV. The influence of an excessively long loop of the lead irritating the tricuspid valve on the development of LDTVD was already presented in an earlier study of the authors [24]. The loop located in the tricuspid ostium, by persistent valve opening, contributes to the development of a significant TR, at the same time it grows into the tricuspid apparatus, often causing TV stenosis.

Several earlier reports have shown an influence of the time after CIED implantation on the development of LDTVD [7,15,16,17,25]. In the present study, the effect of duration of the lead in the heart has not been proven, but it should be taken into account that mean lead dwell time in whole study population was very long (95.36 ± 67.10 months), and in the group of patients with LDTVD, it was significantly longer (107.73 ± 68.90; *p* = 0.058). Undoubtedly, an expression of the influence of time on the development of LDTVD in the present study is the demonstration of the predictive role of the growth of leads to the structures of the heart.

Some studies document a close relationship between LDTVD and lead location that causes anterior or posterior leaflets impingement [26,27]. The course of the lead through the TV is also closely related to the target site of right ventricular pacing. In the formerly common apical pacing, the axial course through the TV is more favorable for a better cooptation of the leaflets. In turn, the currently more popular septal pacing may predispose to the occurrence of LDTVD through a much less axial course and a potential conflict with the chordae tendinae. In the present study, the tendency to more often LDTVD in patients with non-apical location of the lead tip was observed (*p* = 0.09), meanwhile, previous studies show completely different results Some reports have documented the unfavorable effect of septal pacing on the development of LDTVD [8,28] while others suggest the opposite—adverse effect of apical pacing, probably related to contraction dyssynchrony [9,29].

The present study also looked at the possibility of improving tricuspid valve function in patients with LDTVD after TLE. The reduction of TR after TLE was demonstrated in 35.29% of patients and was found more frequently in elderly patients with ischemic heart disease, qualified for TLE for prophylactic indications, and in patients with a smaller right ventricular size. Reports on the improvement of valve function in patients with LDTVD after TLE are sparse, in fact only the authors’ previous analysis shows a significant reduction in TR [24], Other studies do not confirm the improvement of TV function in these patients, attributing it to the dilation of the tricuspid valve annulus persisted following lead removal [30,31].

The relationship between LDTVD and worse survival is well known, and worsening of TR has been identified as an independent risk factor for mortality [15,16,32,33]. Analysis of long-term survival in the present study showed higher mortality in patients with LDTVD, but for the first time it was documented that this only applied to patients who did not improve after TLE. Patients with LDTVD who showed a decrease in TR after TLE had the best survival after approximately 5 years of follow-up.

## 5. Conclusions

Lead dependent tricuspid dysfunction is diagnosed in 4.44% of patients referred for lead extraction due to different reasons and only in 37.82% LDTVD is main TLE indications. The most common mechanism of LDTVD is propping upward or clumping down the leaflet by the lead. Improvement of tricuspid valve function after TLE in population with very long lead dwell time (above 8 years) is observed in 35.29% of patients. The risk factors for LDTVD are primarily related to the course of the lead and its adhesion to the heart structures especially in the women. The influence of other clinical factors (valvular left heart disease, atrial fibrillation, heart failure resulting in higher pulmonary artery systolic pressure) results in dilation of the right ventricle and secondary favoring the dilatation of the valve annulus exacerbating its regurgitation. Patients with LDTVD have a worse long-term survival, but the improvement in valve function following TLE contributes to a significant reduction in mortality.

## Figures and Tables

**Table 1:** Basic information about 119 patients with confirmed LDTVD.

**Prelininary and verifired diagnosis of LDTVD**			**Effect of TLE on severity of TR in pts with LDTVD impression of echocardioigraphist**		
Preliminary diagnosis of LDTVD	125	4.90%	Insignificant	73	61.34%
Confirmed diagnosis of LDTVD	119	4.44%	Perceptible	33	27.73%
All patients	2678	100.00%	Significant	13	10.92%
**Main/predominant Indications for lead extraction in patient with LDTVD**			All LDTVD patients	119	100.00%
Symptomatic lead dependent TV dysfunction	45	37.82%			
Lead damage/dysfunction (lead replacement)	39	32.78%	**Changes of degree of TR after TLE (degrees)**		
Systemic infection or local or mixed infection	21	17.65%	No change (the same)	77	64.71%
Upgrading, downgrading, prevention of lead abandonment	11	9.24%	Reduction of TR for 1 degree	38	31.93%
Recapture venous access (symptomatic occlusion, lead replacement/upgrading)	3	2.52%	Reduction of TR for 2 degrees	4	3.36%
All LDTVD patients	119	100.00%	All LDTVD patients	119	100.00%
**Mechanism of TV dysfunction (partial immobilisation of the leaf or irritation causing degeneration)**			Average right ventricular lead dwell time	104.57	SD 69.8
Propping upward the leaflet by the lead	45	37.82%	**LDTVD and cardiac surgery after TLE**		
Drawing down f the leaflet by the lead (immobilisation)	57	47.90%	Indication reached—observation	22	18.49%
Impingement of the leaflet by the lead presence (irritation)	3	2.52%	No indication—observation only	74	62.19%
Perforation of the leaflet with the lead	3	2.52%	Referred for TV plastic	18	15.13%
Connection of lead with the lead with scar	11	9.24%	Not considered (contraindication, lack of agreement)	5	4.20%
All LDTVD patients	119	100.00%	ALL patients with LDTVD	119	100.00%

Abbreviations: LDTVD—lead dependent tricuspid valve dysfunction, TLE—transvenous lead extraction, TV—tricuspid valve, TR- tricuspid regurgitation.

**Table 2:** Clinical data-classification according to the occurrence of LDTVD.

Patient-Related Potential Risk Factors of LDTVD	LDTVD (All Patients with LDTVD)		NO LDTVD (Control Group)		
Number of patients/number of the group	119	1	2559	2	*p*
Presented values	Count/average	%/Sd	Count/average	%/Sd	1 vs. 2
Patient’s age during TLE	68.09	15.05	66.90	14.52	0.087
Patient’s age during first system implantation	58.43	18.96	58.32	16.18	0.485
Sex (% of female patients)	67	56.30%	995	38.88%	0.001
Baseline heart diseases: IHD, MI	69	57.98%	1494	58.38%	0.995
Baseline heart diseases: primary cardiomyopathy	13	10.92%	343	13.40%	0.562
Baseline heart diseases: valvular heart disease	10	8.40%	59	2.31%	0.001
Baseline heart diseases: post-inflammatory, congenital, channelopathies, neurocardiogenic, unknown	27	22.69%	662	25.87%	0.566
NYHA class I–IV	2.20	0.80	1.84	0.67	<0.001
AF permanent	50	42.02%	565	22.08%	0.001
Hypertension	66	55.46%	1487	58.11%	0.777
Diabetes (any)	21	17.65%	534	20.87%	0.515
Renal failure (any)	34	28.57%	538	21.02%	0.052
Valvular implant presence	83	18.49%	156	6.10%	0.001
Mechanical valve presence	15	12.61%	15	3.59%	0.001
Previous sternotomy	24	20.17%	359	14.03%	0.07
Long-term anticoagulation	73	61.35%	1000	39.08%	0.001
Charlson’s index (points)	4.99	3.56	4.83	3.69	0.352

Abbreviations: AF—atrial fibrillation, IHD—ischaemic heart disease, MI—myocardial infarction, LDTVD—lead dependent tricuspid valve dysfunction, NYHA class—New York Hear Association class, TLE—transvenous lead extraction. Valvular implant: mechanical valve, biological valve, mitral o tricuspid ring.

**Table 3:** Indication, system and history of pacing -related factors. Classification according to the occurrence of LDTVD.

	LDTVD (All Patients with LDTVD)		NO LDTVD (Control Group)		
Number of Patients Number of the Group	119	1	2559	2	*p*
Presented Values	Count/Average	%/ Sd	Count/Average	%/Sd	1 vs. 2
**TLE Indications**					
LRIE certain with or without pocket infection	13	10.92%	458	17.90%	0.078
LRIE probable with or without pocket infection	4	3.36%	159	6.21%	0.279
Local/isolated pocket infection	4	3.36%	206	8.05%	0.099
All infections	21	17.65%	823	32.16%	0.002
Non-infectious prophylactic indications	5	4.20%	86	3.36%	0.788
Non-infectious therapeutic indications	93	78.15%	1650	64.48%	0.002
All non-infectious indications	98	82.35%	1736	67.84%	0.001
**System and history of pacing**					
Device type—PM (AAI, VVI, DDD, CRT-P)	100	84.03%	1786	69.79%	0.001
Device type—ICD (VVI, DDD)	9	7.56%	577	22.55%	0.001
Device type—CRT-D	10	8.40%	196	7.66%	0.865
Number of leads in the system before TLE	1.84	0.70	1.82	0.62	0.971
Presence of abandoned lead before TLE	16	13.45%	270	10.55%	0.363
Number of abandoned leads before TLE	0.21	0.60	0.14	0.43	0.636
Number of leads in the heart before TLE	2.03	0.82	1.95	0.73	0.459
4 and >4 leads before TLE	7	5.88%	73	2.85%	0.096
One ICD lead before TLE	18	15.13%	762	29.78%	0.001
2 or more ICD leads before TLE	2	1.68%	18	7.03%	0.493
Apical RV lead location (lead analysis)	101	41.91%	2058	41.39%	0.166
Out of apical (septal, outfow tract, anterior wall) RV lead location (lead analysis)	30	12.45%	483	9.71%	0.092
Previous TLE in history	8	6.72%	135	5.28%	0.603
Upgrading or downgrading with lead abandonment	11	9.24%	158	6.17%	0.229
Excessive long lead loop in the atrium (fluoroscopy)	18	15.13%	311	12.15%	0.374
Excessive lead loop crossing TV or in the ventricle (fluoroscopy)—A	24	20.17%	116	4.53%	0.001
Fluoroscopic impression of lead collision with TV (without loop) to tense or to long—B	25	21.01%	15	0.59%	0.001
Fluoroscopic impression of lead loop collision with TV—C	25	21.01%	115	4.49%	0.001
All lead’s collision with TV (A + B + C)	74	62.19%	246	9.61%	0.001
Dwell time of oldest one lead in the patient before TLE	116.82	81.90	103.73	75.64	0.106
Mean lead dwell time (in the patient) before TLE (in months)	107.73	68.90	95.83	66.98	0.058

Abbreviations: CRT—cardiac resynchronization therapy, ICD—implantable cardioverter defibrillator, LRIE—lead related infective endocarditis, MI—myocardial infarction, LDTVD—lead dependent tricuspid valve dysfunction, PM—pacemaker, RV—right ventricle, TLE—transvenous lead extraction, TV—tricuspid valve.

**Table 4:** TLE procedure complexity, efficacy, complications, outcomes and long-term mortality after TLE. Classification according to the occurrence of LDTVD.

TLE Procedure Complicity, Efficacy, Complications, Outcomes and Long-Term Mortality after TLE	LDTVD (All Patients with LDTVD)		NO LDTVD (Control Group)		
Number of Patients Number of the Group	119	1	2559	2	*p*
Presented Values	Count/Average	%/Sd	Count/Average	%/Sd	1 vs. 2
**TLE procedure complexity**					
Procedure duration (sheath to sheath)	29.66	35.90	14.44	21.60	0.242
Average time of single lead extraction (sheath-to sheath/number of extracted leads)	11.05	17.24	8.64	12.03	0.512
Technical problem during TLE (any)	29	24.37%	531	20.75%	0.356
Number of big technical problems	1.71	1.15	1.34	0.65	0.153
One technical problem only	16	13.45%	322	12.58%	0.842
Two or more technical Problems	12	10.08%	113	4.42%	0.007
**Utility of additional tools**					
Evolution (old and new) or TighRail	6	5.04%	38	1.49%	0.008
Lasso catheter/snare	9	7.56%	94	3.67%	0.050
Basket catheter	1	0.84%	19	0.74%	0.683
**TLE efficacy and complications**					
Major complications (any)	1	0.84%	50	1.95%	0.613
Hemopericardium	1	0.84%	28	1.09%	0.834
Haemothorax	0	0.00%	3	0.31%	0.298
Tricuspid valve damage (significant) during TLE	0	0.00%	17	0.66%	0.771
Rescue cardiac surgery	0	0.00%	23	0.90%	0.604
Minor complications (any)	14	11.76%	204	7.98%	0.172
Death procedure related (intra, post-procedural)	0	0.00%	0	0.00%	N
Death indication-related (intra, post-procedural)	0	0.00%	1	0.04%	0.026
Partial radiological success (remained tip or < 4 cm lead fragment)	10	8.40%	93	3.63%	0.014
Full clinical success	118	99.16%	2504	97.85%	0.17
Full procedural success	108	90.76%	2446	95.58%	0.191
**Long-term mortality after TLE**					
Alive during 1658 ± 1203 (1–5519) days of follow up	78	65.55%	1796	70.18%	0.329
Death during all 1658 ± 1203 (1–5519) days of follow up	41	34.45%	763	29.82%	0.329

Abbreviations: LDTV—lead dependent tricuspid valve dysfunction, TLE—transvenous lead extraction.

**Table 5:** Echocardiographic findings/abnormalities. Classification according to the occurrence of LDTVD.

Echocardiographic Findings/Abnormalities Recorded in Patients with or without LDTVD	LDTVD (All Patients with LDTVD)		NO LDTVD (Control Group)		
Number of Patients Number of the Group	119	1	2559	2	*p*
Presented Values	Count/Average	%/Sd	Count/Average	%/Sd	1 vs. 2
**Echocardiography before and after TLE**					
Average LVEF	48.58	13.27	49.53	15.46	0.153
Mitral regurgitation (significant)	24/112	21.43%	352/2528	13.92%	0.037
PASP (mmHg)	40.73	13.41	30.49	13.16	0.001
RV diameter (mm)	35.51	7.65	31.24	6.00	0.001
**Tricuspid Regurgitation before TLE**					
Non-significant (0, 1, 2 grade )	14/119	11.77%	2123/2553	83.16%	0.001
Significant (3 grade)	48/119	40.34%	360/2553	14.10%	0.001
Severe (4 grade)	57/119	47.90%	70/2553	2.74%	0.001
**Any shadows on the leads before TLE**					
Any shadows on leads before TLE	53/119	52.94%	1264/2554	49.39%	0.336
Connecting tissue surrounding the lead	7/119	5.88%	262/2557	10.25%	0.164
Blood cloth on the lead	11/119	9.24%	164/2557	6.41%	0.336
Vegetation-like mass	1/119	0.84%	107/2557	4.18%	0.116
Thicker lead	26/119	21.85%	470/2557	18.38%	0.406
Vegetation	16/119	13.45%	450/2556	17.61%	0.296
Strong connective tissue scar connection of the lead with heart structures (any)	28/115	24.35%	315/2498	12.61%	0.001
Strong connective tissue scar connection of the lead with tricuspid apparatus	17/119	14.29%	124/2557	4.85%	0.001
Strong connective tissue scar connection of the lead with RA wall	11/119	9.24%	99/2557	3.87%	0.008
Strong connective tissue scar connection of the lead with SVC	5/119	4.20%	104/2557	4.07%	0.869
Strong connective tissue scar connection of the lead with RV wall	14/119	11.77%	157/2557	6.14%	0.073
**Loops of the leads**					
Excessive loops of the leads in the heart (any)/ECHO	43/119	36.13%	450/2558	17.59%	0.001
Excessive loop in the RA	27/119	22.69%	331/2558	12.94%	0.001
Excessive loop in the TV	27/119	22.69%	94/2558	2.68%	0.001
Excessive loop in the RV	20/119	16.81%	124/2558	4.85%	0.001

Abbreviations: LVEF—left ventricular ejection fraction, LDTVD—lead dependent tricuspid valve dysfunction, PASP—pulmonary artery systolic pressure, RA—right atrium, RV—right ventricle, SVC—superior vena cava, TLE—transvenous lead extraction, TV—tricuspid valve.

**Table 6:** Factors connected with LDTVD presence. Results of uni- and multi-variable logistic regression analysis.

19 LDTVD vs. Other (2559)	Univariate Analysis			Multivariate Analysis		
	OR	95% CI	*p*	OR	95% CI	*p*
Patient’s age during TLE (by year)	1.008	0.995–0.021	0.239			
Female gender	1.927	1.327–1.797	<0.001	2.441	1.514–3.937	<0.001
Baseline heart diseases: valvular heart disease	3.248	1.860–1.669	<0.001	2.755	1.153–6.580	0.022
NYHA class (by 1)	2.092	1.609–2.720	<0.001	2.060	1.443–2.940	<0.001
PASP (by 1 mm Hg)	1.047	1.035–1.060	<0.001	1.030	1.014–1.046	<0.001
RV diameter (by 1 mm)	1.099	1.073–1.126	<0.001	1.079	1.043–1.116	<0.001
Permanent AF	2.555	1.750–3.730	<0.001	1.776	0.985–3.201	0.056
Mitral valve insufficiency	1.897	1.214–2.963	0.004	1.038	0.586–1.840	0.879
Renal failure (any)	1.447	0.956–2.191	0.081	1.058	0.631–1.773	0.831
Valvular implant presence	2.801	1.633–4.804	<0.001	1.045	0.431–2.534	0.922
Previous sternotomy	1.377	0.855–2.216	0.187			
Long term anticoagulation	2.254	1.547–3.284	<0.000	1.152	0.634–2.092	0.643
Presence of pacing leads only (AAI VVI VDD DDD CRTP)	2.116	1.321–3.390	0.002	1.812	1.001–3.279	0.050
Presence of HV lead(s) (without CRTD)	0.406	0.238–0.693	<0.001			
≥4 leads before TLE	1.848	0.788–4.334	0.158			
Mean lead dwell time before TLE (by year)	1.030	0.999–1.063	0.062	0.969	0.925–1.015	0.184
* Lead(s) collision with TV (including excessive loop (s) of lead (s))	12.765	8.506–19.16	<0.001	15.283	9.101–25.663	<0.001
* Excessive loops of the leads in the heart (any)/ECHO	2.790	1.878–4.143	<0.001			
Excessive loop in the RA/ECHO	2.031	1.290–3.197	0.002			
Excessive loop in the TV/ECHO	7.630	4.701–12.385	<0.001			
Excessive loop in the RV/ECHO	4.034	2.382–6.830	<0.001			
Strong connective tissue scar connection of the lead with heart structures (any)	2.489	1.605–3.861	<0.001			
Strong connective tissue scar connection of the lead with tricuspid apparatus	3.397	1.938–5.954	<0.001	2.004	0.958–4.196	0.065
Strong connective tissue scar connection of the lead with RA wall	2.934	1.520–5.663	<0.001	3.601	1.639–7.912	<0.001
Strong connective tissue scar connection of the lead with RV wall	2.313	1.289–4.151	0.005	2.151	0.992–4.663	0.052

* Due to the result of the two-factor regression analysis for the multivariate model, the variable “Lead (s) collision with TV (including excessive loop (s) of lead (s))” was selected, ignoring the change “excessive loops of the leads in the heart (any)/ECHO”. OR for “leads collision” = 14.942; *p* < 0.001 and OR for “excessive loops” = 0.750; *p* = 0.311. Abbreviations: AF—atrial fibrillation, CRT—cardiac resynchronization therapy, LDTVD—lead dependent tricuspid valve dysfunction, HV—high voltage lead, PASP—pulmonary artery systolic pressure, RA—right atrium, RV—right ventricle, TLE—transvenous lead extraction, TV—tricuspid valve.

## Data Availability

Readers can access the data supporting the conclusions of the study at www.usuwanieelektrod.pl (accessed on 2 November 2021).

## Supplementary Materials

The following supporting information can be downloaded at: https://www.mdpi.com/article/10.3390/jcm11010089/s1, Table S1: Clinical data. Classification according to the influence of TLE on LDTVD; Table S2: Indication, system and history of pacing-related factors. Classification according to the influence of TLE on LDTVD; Table S3: TLE procedure complexity, efficacy, complications, outcomes and long-term mortality after TLE. Classification according to the influence of TLE on LDTVD; Table S4: Echocardiographic findings/abnormalities. Classification according to the influence of TLE on LDTVD.
