# Supplementary material for: Lead Dependent Tricuspid Valve Dysfunction-Risk Factors, Improvement after Transvenous Lead Extraction and Long-Term Prognosis
Source: J Clin Med. 2021 Dec 24;11(1):89. doi: 10.3390/jcm11010089 (PMC8745716; doi:10.3390/jcm11010089)
Supplement: Supplementary file 1 [file jcm-11-00089-s001.zip › jcm-1491916-supplementary.pdf]

Supplementary file

Lead dependent tricuspid valve dysfunction- risk factors, improvement after transvenous lead extraction and long-term prognosis

Table S1. Clinical data. Classification according to the the influence of TLE on LDTVD

	LDTVD improvement after TLE		LDTVD Lack of improvement after TLE			All examined patients (2678)	
**Number of patients / number of the group**	**42**	**3**	**77**	**4**	**P**	**2678**	**5**
**Presented values**	Count / average	**% / Sd**	Count / average	**% / Sd**	**3 vs 4**	Count / average	**% / Sd**
**Patient-related potential risk factors**							
Patient's age during TLE	68,93	17,72	67,64	13,50	0,049	66,95	14,55
Patient's age during first system implantation	60,65	18,57	57,27	16,02	0,028	58,32	16,21
Sex (% of female patients)	27	64,29%	40	51,95%	0,27	1062	39,66%
Baseline heart diseases: IHD, MI	30	71,43%	39	50,65%	0,05	1563	58,36%
Baseline heart diseases: primary cardiomyopathy	4	9,52%	9	11,69%	0,96	356	13,29%
Baseline heart diseases: valvular heart disease	0	0,00%	10	12,99%	0,036	69	2,58%
Baseline heart diseases: post-inflammatory .congenital, channelopathies, neurocardiogenic, unknown	6	19,05%	19	24,68%	0,274	689	25,73%
NYHA class I-IV	2.17	0.73	2.21	0.84	0.74	1.86	0.68
AF permanent	16	38,10%	34	44,16%	0,66	615	22,97%
Hypertension	24	57,14%	42	54,55%	0,94	1553	57,99%
Diabetes (any)	10	23,81%	11	14,29%	0,29	555	20,72%
Renal failure (any)	10	23,81%	24	31,17%	0,52	572	21,36%
Valvular implant presence	3	7,14%	19	24,68%	0,035	178	6,65%
Mechanical valve presence	3	7,14%	12	15,58%	0,3	107	4,00%
Previous sternotomy	4	9,52%	20	25,97%	0,058	383	14,30%
Long-term anticoagulation	25	59,52%	48	62,34%	0,92	1073	40,07%
Charlson's index (points)	5,62	4,13	4,65	3,19	0,21	4,84	3,68

Table S2. Indication, system and history of pacing -related factors. Classification according to the influence of TLE on LDTVD

	LDTVD improvement after TLE		LDTVD Lack of improvement after TLE			All examined patients (2678)	
**Number of patients number of the group**	**42**	**3**	**77**	**4**		**2678**	**5**
**Presented values**	**Count / average**	**% / Sd**	**Count / average**	**%/ Sd**	**3 vs 4**	**Count / average**	**%/ Sd**
**TLE indications**							
LRIE certain with or without pocket infection	0	0,00%	13	16,88%	0,012	471	17,59%
LRIE probable with or without pocket infection	2	4,76%	2	2,60%	0,925	163	6,09%
Local / isolated pocket infection	3	7,14%	1	1,30%	0,247	210	7,84%
All infections	5	11,91%	16	20,78%	0,336	844	31,52%
Non-infectious prophylactic indications	34	80,95%	2	2,60%	0,001	91	3,40%
Non-infectious therapeutic indications	0	0,00%	59	76,62%	0,001	1743	65,09%
All non-infectious indications	34	80,95%	61	79,22%	0,989	1834	68,48%
**System and history of pacing**							
Device type - PM (AAI, VVI, DDD, CRT-P)	35	85,71%	64	83,12%	0,821	1886	70,43%
Device type - ICD (VVI, DDD)	4	9,52%	5	6,49%	0,814	585	22,88%
Device type - CRT-D	2	4,76%	8	10,39%	0,477	206	7,69%
Number of leads in the system before TLE	1,83	0,58	1,84	0,76	0,647	1,83	0,63
Presence of abandoned lead before TLE	5	11,91%	11	14,29%	0,716	286	10,68%
Number of abandoned leads before TLE	0,12	0,33	0,260	0,70	0,599	0,14	0,44
Number of leads in the heart before TLE	1,95	0,58	2,078	0,93	0,647	1,96	0,73
4 and > 4 leads before TLE	1	2,38%	6	7,79%	0,429	80	2,99%
One ICD lead before TLE	5	11,91%	13	16,88%	0,648	780	29,13%
2 or more ICD leads before TLE	1	2,38%	1	1,30%	0,759	29	1,08%
Apical RV lead location (lead analysis)	32	39,51%	69	43,13%	0,092	2159	41,42%
Out of apical (septal, outfow tract, anterior wall) RV lead location (lead analysis)	12	14,82%	18	11,25%	0,687	513	9,84%
Previous TLE in history	3	7,14%	4	5,20%	0,981	143	5,35%
Upgrading or downgrading with lead abandonment	4	9,52%	7	9,09%	0,819	169	6,31%
Excessive long lead loop in the atrium (fluoroscopy)	9	21,43%	9	11,69%	0,250	329	12,48%
Excessive lead loop crossing TV or in the ventricle (fluoroscopy) A	7	16,67%	17	22,08%	0,643	140	5,23%
Fluoroscopic impression of lead collision with TV (without loop) to tense or to long B	11	26,19%	14	18,18%	0,430	40	1,49%
Fluoroscopic impression of lead loop collision with TV C	8	19,05%	17	22,08%	0,879	140	5,23%
All lead's collision with TV (A+B+C)	26	61.91%	48	62.34%	0.898	320	11.95%
Dwell time of oldest one lead in the patient before TLE	100,69	65,430	125,623	88,780	0,335	104,313	75,96
Mean lead dwell time (in the patient) before TLE (in months)	94,57	56,02	114,91	74,36	0,325	95,36	67,10

Abbreviations: CRT- cardiac resynchronization therapy, ICD- implantable cardioverter defibrillator, LRIE- lead related infective endocarditis, MI- myocardial infarction, LDTVD- lead dependent tricuspid valve dysfunction, PM- pacemaker, RV- right ventricle, TLE- transvenous lead extraction, TV- tricuspid valve,

Table S3. TLE procedure complexity, efficacy, complications, outcomes and long-term mortality after TLE. Classification according to the influence of TLE on LDTVD.

**TLE procedure complicity, efficacy, complications, outcomes and long-term mortality after TLE**	**LDTVD improvement after TLE**		**LDTVD Lack of improvement after TLE**			**All examined patients (2678)**	
**Number of patients number of the group**	**42**	**3**	**77**	**4**	**P**	**2678**	**5**
**Presented values**	**Count / average**	**% / Sd**	**Count / average**	**% / Sd**	**3 vs 4**	**Count / average**	**% / Sd**
**TLE procedure complexity**							
Procedure duration (sheath to sheath)	14,00	15,19	24,30	42,877	0,800	14,75	22,45
Average time of single lead extraction (sheath-to sheath / number of extracted leads)	7,675	7,157	12,883	20,590	0,998	8,576	12,313
Technical problem during TLE (any)	7	16,67%	22	28,57%	0,222	560	20,91%
Number of big technical problems	1,43	1,79	1,82	1,25	0,302	1,365	0,70
One technical problem only	5	11,91%	11	14,29%	0,934	338	12,62%
Two or more technical Problems	2	4,76%	10	12,99%	0,269	125	4,45%
**Utility of additional tools**							
Evolution (old and new) or TighRail	1	2,38%	5	6,49%	0,988	44	1,64%
Lasso catheter / snare	0	0,00%	7	9,09%	0,108	103	3,85%
Basket catheter	2	4,76%	1	1,30%	0,589	20	0,75%
**TLE efficacy and complications**							
Major Complications (any)	0	0,00%	1	1,30%	0,757	51	1,90%
Hemopericardium	0	0,00%	1	1,30%	0,757	29	1,12%
Haemothorax	0	0,00%	0	0,00%	N	3	0,11%
Tricuspid valve damage (significant) during TLE	0	0,00%	0	0,00%	N	17	0,64%
Rescue cardiac surgery	0	0,00%	0	0,00%	N	24	89,60%
Minor complications (any)	3	7,14%	11	14,29%	0,391	218	8,15%
Death procedure related (intra, post-procedural)	0	0,00%	0	0,00%	N	0	0,00%
Death indication-related (intra, post-procedural	0	0,00%	0	0,00%	N	1	0,04%
Partial radiological success (remained tip or < 4 cm lead fragment)	1	2,38%	9	11,69%	0,161	103	3,85%
Full Clinical Success	42	100,00%	76	98,70%	0,757	2622	97,91%
Full Procedural Success	41	97,62%	67	87,01%	0,115	2554	95,37%
**Long-term mortality after TLE**							
Alive during 1658 ± 1203 (1 – 5519) days of follow up	34	80.95%	44	57.14%	0.016	1874	69.98%
Death during all 1658 ± 1203 (1 – 5519) days of follow up	8	19.05%	33	42.86%	0.016	804	30.02%

Abbreviations: LDTVD- lead dependent tricuspid valve dysfunction, TLE- transvenous lead extraction

Table S4. Echocardiographic findings / abnormalities. Classification according to the influence of TLE on LDTVD

Echocardiographic findings / abnormalities recorded in patients with or without LDTVD	LDTVD improvement after TLE		LDTVD Lack of improvement after TLE			All examined patients (2678)	
**Number of patients number of the group**	**42**	**3**	**77**	**4**		**2678**	**5**
**Presented values**	**Count / average**	**% /Sd**	**Count / average**	**% /Sd**	**3 vs 4**	**Count / average**	**% / Sd**
**Echocardiography before and after TLE**							
Average LVEF	46,93	13,88	49,48	12,93	0,532	49,48	15,37
Mitral regurgitation (significant)	10/42	23,81%	14/70	20,00%	0,576	376/2640	14,24%
PASP (mmHg)	42,46	12,51	39,70	13,84	0,354	30,95	13,34
RV diameter (mm)	33,83	6,20	36,60	8,08	0,081	31,43	6,14
**Tricuspid Regurgitation before TLE**							
Non-significant (0,1,2 grade)	0/42	0,00%	14/77	18,18%	0,008	2137/2672	79,99%
Significant (3 grade)	18/42	42,86%	30/77	38,96%	0,827	408/2672	15,27%
Severe (4 grade)	24/42	57,14%	33/77	42,86%	0,217	127/2672	4,75%
**Any shadows on the leads before TLE**							
Any shadows on leads before TLE	20/42	47,62%	43/77	55,84%	0,546	1327/2673	49,55%
Connecting tissue surrounding the lead	2/42	4,76%	5/77	6,49%	0,981	269/2676	10,05%
Blood cloth on the lead	3/42	7,14%	8/77	10,39%	0,8	175/2676	6,50%
Vegetation-like mass	1/42	2,38%	3/77	3,90%	0,925	111/2676	4,15%
Thicker lead	11/42	26,19%	15/77	19,48%	0,539	496/2676	18,54%
Vegetation	2/42	4,76%	14/77	18,18%	0,077	466/2675	17,42%
Strong connective tissue scar connection of the lead with heart structures (any)	7/41	16,67%	21/74	28,38%	0,131	343/2613	13,13%
Strong connective tissue scar connection of the lead with tricuspid apparatus	2/42	4,76%	15/77	19,48%	0,055	141/2676	5,27%
Strong connective tissue scar connection of the lead with RA wall	5/42	11,91%	6/77	7,79%	0,683	110/2676	4,11%
Strong connective tissue scar connection of the lead with SVC	0/42	0,00%	5/77	6,49%	0,227	109/2676	4,07%
Strong connective tissue scar connection of the lead with RV wall	6/42	14,29%	8/77	10,39%	0,824	171/2676	6,39%
**Loops of the leads**							
Excessive loops of the leads in the heart (any) / ECHO	16/42	38,10%	27/77	35,07%	0,957	493/2677	18,42%
Excessive loop in the RA	10/42	23,81%	17/77	22,08%	0,989	358/2677	13,37%
Excessive loop in the TV	10/42	23,81%	17/77	22,08%	0,989	121/2677	4,52%
Excessive loop in the RV	7/42	16,67%	13/77	16,88%	0,821	144/2677	5,38%

Abbreviations: LVEF- left ventricular ejection fraction, LDTVD- lead dependent tricuspid valve dysfunction, PASP- pulmonary artery systolic pressure, RA- right atrium, RV- right ventricle, SVC- superior vena cava, TLE- transvenous lead extraction, TV- tricuspid valve
